# General practice veterinarians’ attitudes towards avian influenza: A COM‐B analysis of barriers to backyard poultry treatment

**DOI:** 10.1002/vetr.70173

**Published:** 2025-12-22

**Authors:** Sol Elliott, Sarah Jewitt, Emma McClaughlin, Matthew Smallman‐Raynor, Sioux Fisher, Michael Clark, Rachael Tarlinton

**Affiliations:** ^1^ Farm Gate Vets Lancaster UK; ^2^ School of Veterinary Medicine and Science University of Nottingham Sutton Bonington UK; ^3^ School of Geography University of Nottingham Nottingham UK; ^4^ School of English University of Nottingham Nottingham UK; ^5^ One Virology, The Wolfson Centre for Global Virus Research, School of Veterinary Medicine and Science University of Nottingham Sutton Bonington UK

**Keywords:** backyard poultry, COM‐B model, general practice veterinarians, highly pathogenic avian influenza

## Abstract

**Background:**

The recent expansion of highly pathogenic avian influenza (HPAI) H5N1 to non‐avian species in the United States has intensified public health‐related concerns. In Great Britain, low veterinarian confidence in seeing and treating birds creates potential barriers to HPAI diagnosis and reporting.

**Methods:**

An online survey explored general practice (GP) veterinarians’ confidence in and barriers to treating avian species and understanding HPAI control measures. The Capability, Opportunity, Motivation ‐ Behaviour (COM‐B) behaviour change model was used to identify barriers to treating birds and diagnosing HPAI.

**Results:**

The survey generated 72 useable responses. Only 6% reported feeling fairly/very confident seeing birds, over 83% lacked confidence in ruling out HPAI as a differential diagnosis and 17.1% were unsure how to advise clients who suspected HPAI in their flocks.

**Limitations:**

The survey required an internet connection and some technical literacy. The sample size is relatively small and may over‐represent veterinarians who have more confidence with poultry.

**Conclusions:**

GP veterinarians play critical roles in triaging, reporting and controlling HPAI. Behaviour change frameworks such as COM‐B can facilitate the identification of interventions with the potential to address barriers to accurate HPAI diagnosis and reporting in Great Britain and beyond. However, these often require national‐level rather than (or in addition to) individual‐level action.

## INTRODUCTION

Highly pathogenic avian influenza (HPAI) H5N1 has recently expanded its host range with increasing spillover to mammals, generating concern about threats to public health.^1^, ^2^, ^3^, ^4^, ^5^, ^6^, ^7^ Although HPAI H5N1 circulation in Great Britain remains largely confined to birds, significant outbreaks in 2021‒2022, 2022‒2023 and 2024‒2025 plus the discovery of H5N1 in a sheep^7^ have raised fears about potential threats to food production and human health.

As the Royal College of Veterinary Surgeons has not made companion poultry a compulsory aspect of the curriculum,^8^ there are no means to assess competency in veterinarians dealing with these birds, and while the number of British veterinarians who see backyard poultry is unknown, most are (occasionally) asked to assess an owned or wild bird. Although small‐scale poultry keepers view veterinarians as trustworthy and are likely to seek HPAI advice from them,^8^, ^9^, ^10^, ^11^, ^12^, ^13^, ^14^ general practice (GP) veterinarians often lack confidence with poultry and competency in triaging companion poultry in a timely manner, causing delayed reporting of suspected HPAI cases.^8^, ^12^, ^15^ Previous studies have identified low confidence as a behavioural barrier^16^, ^17^ yet relationships between veterinarian confidence and training in poultry medicine, particularly backyard poultry, are poorly understood. Little is known about veterinarians’ motivation or ability to treat avian species or knowledge of HPAI control and reporting measures. Understanding these gaps will support training providers to develop further resources for veterinarians and enhance knowledge of HPAI control measures.

**FIGURE 1:**
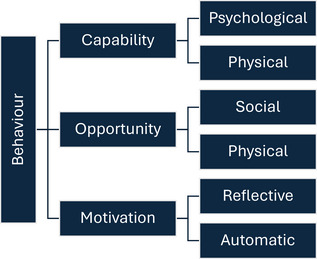
Diagram of COM‐B plus subcomponents.Adapted from Michie et al. 2011.^18^

Behavioural and cognitive psychology help to reveal the psychological processes behind behaviour and inform effective behaviour change strategies (here, veterinarians feeling prepared and willing to see backyard poultry). The behaviour change model, Capability, Opportunity, Motivation ‐ Behaviour (COM‐B)^18^ provides a framework for understanding the conditions required for (and barriers to) behaviour change. The model links behaviour change to three interrelated components: (i) capability—the knowledge, skills and psychological or physical capacity to perform desired behaviours; (ii) opportunity—the external factors enabling individuals to perform behaviours, including systems or environments that hinder or facilitate action^18^; and (iii) motivation—the internal processes (including desires, beliefs, emotions or habits) driving individuals to act.^18^ These components are further divided into two subcomponents (Figure 1).

Capability subcomponents comprise ‘psychological’ (cognitive skills and knowledge) and ‘physical’ (skills including dexterity and coordination) attributes necessary to carry out behaviours. Opportunity subcomponents are ‘social’ (norms and environments influencing the desire to perform behaviours) and ‘physical’ (environmental factors including resources and time). Motivation is divided into ‘automatic’ (spontaneous emotions, needs and desires that influence behaviour) and ‘reflective’ (planned actions brought about by previous self‐evaluation).

COM‐B has been widely used to understand barriers and enablers faced by individuals in adopting health‐related behaviours,^19^, ^20^ including those related to cattle and sheep lameness, pet obesity and antibiotic usage in production animals,^21^, ^22^, ^23^, ^24^, ^25^ with ‘COM’ components used to identify small‐scale poultry keepers’ compliance with HPAI prevention measures.^11^

This paper presents the results of an online survey of GP veterinarians in Great Britain, who occasionally see poultry but whose caseloads are not predominantly avian focused. Using COM‐B to identify key barriers and potential enablers for improving confidence with avian species, it recommends additional support (e.g., Continuing Professional Development (CPD) opportunities, veterinary school training, guidance documents) to increase confidence in backyard poultry consultations and improve the care given to these animals. The findings have the potential to inform avian‐related training and guidance to GP veterinarians, support HPAI identification, compliance with national HPAI‐related controls and prompt reporting of suspected notifiable disease cases.

This research builds on a smaller scale study of UK‐based veterinarians’ experiences of treating avian species, handling suspected HPAI cases and views on HPAI control measures. Although this smaller scale study did not focus on GP veterinarians and included some poultry specialists, it highlighted a need for improved training on diagnosing and treating avian species and greater clarity on how veterinary practices should treat birds during HPAI outbreaks.^15^

## MATERIALS AND METHODS

### Survey

To explore veterinarians’ understanding of HPAI control measures, a survey was designed using Microsoft Forms (Appendix 1). It focused on pet/backyard/kept and wild birds and was targeted at GP veterinarians working in private practice, whose caseload was not predominantly avian focused but who may see birds if requested.

Following ethical approval, the draft questionnaire was tested with veterinary colleagues who fit the target criteria and edited to improve clarity and flow. The final survey ran from 19 March 2024 to 8 June 2024 and comprised 19 closed‐text and eight open‐text (Q.2, 6, 8, 14, 15, 18, 20 and 27) questions. Questions were a mixture of open‐ended and multiple‐choice questions, with one Likert scale (Q.24). The survey was advertised via social media (Facebook groups ‘VetWings’ and ‘Veterinary Voices’, Twitter/X and LinkedIn) and distributed in person via QR code at the British Small Animal Veterinary Association (BSAVA) Congress and BVA Live conferences 2024. No identifying information was captured beyond the first part of the practice postcode (Q.2).

Questions regarding practice type and the number/type of birds seen (Q.3–4, Q.9–10) assessed respondents’ caseloads. Information was also collected on awareness and sources of information on HPAI and its control measures (Q.11–14), where respondents believed that their clients obtained HPAI‐related information (Q.23).

To help identify factors that could delay HPAI diagnosis and reporting, information was requested on confidence in seeing and treating birds, avian medicine training, knowledge of HPAI‐related clinical signs, confidence and experiences assessing and advising on a suspected case and any barriers to their practice seeing birds (Q.5–8, Q.15–22). To further measure understanding of HPAI control measures, three ‘true or false’ questions were asked (Q.21), based on British Veterinary Association HPAI‐related guidance.^26^

Veterinarian perspectives on ease of housing order compliance and client demand for vaccination (including the price that clients are thought willing to pay) were also requested (Q.24–26). Finally, Q.27 allowed for optional further comments.

### Data analysis

Closed‐text responses were analysed using simple descriptive statistics and statistical tests of independence. For the latter, respondents were dichotomously classified by practice type (Q.3) according to the treatment (yes/no): small animals only, small animals, farm animals, equids and exotics. Statistical tests of independence were performed between the practices so classified and (i) avian species treated (Q.4) and (ii) confidence in the differential diagnosis of HPAI (Q.16). Statistical tests of independence were performed between self‐rated confidence (Q.5) (classified as ‘low’ [levels 1–2] and ‘medium or high’ [levels 3–5]) and practice type (Q.3), avian species treated (Q.4), prior bird‐specific training (Q.7) and bird numbers seen per year (Q.9–10) in the binary divisions of 1–15 and 16 or more birds. All tests of independence were performed using Fisher's exact test (*α* = 0.05). For open‐text responses, a manual qualitative approach adapted from directed content analysis was employed.^27^ Themes emerging from the data, along with the literature on veterinarian confidence, guided the iterative categorisation of the responses.^15^ Subsequent deductive coding was undertaken using COM‐B components (Figure 1). For Q.15, responses were coded using the 19 clinical signs provided in the UK Government guidance.^28^ Any additional signs provided by respondents were noted. Open‐text responses were assigned to multiple categories where necessary.

## RESULTS

### Caseload and characteristics

**FIGURE 2:**
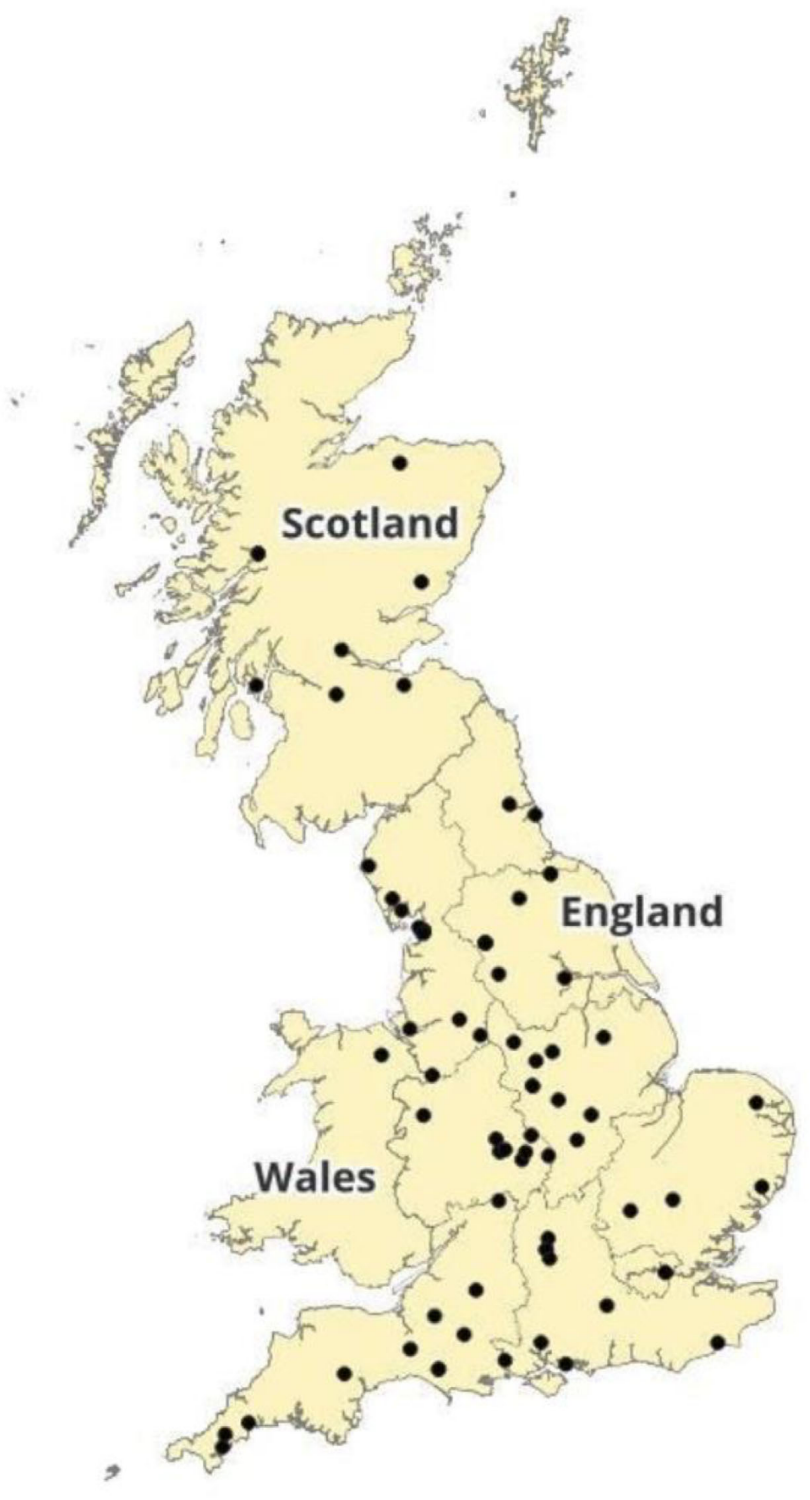
Map of respondents' main practice locations based on first portion of postcode.

Respondents were 79 veterinarians from across Great Britain who confirmed in Q.1 that they were predominantly GP veterinarians, working in private practice, who would describe their caseload as ‘not predominantly avian focused’ but may see pet or backyard poultry if requested.* Six pilot responses and one incomplete response were excluded from the final analysis, leaving 72 usable responses. There was a wide spread of practice postcodes, with most respondents from England (Figure 2). When asked to define their caseload (respondents could select multiple options), over 75% saw small animals, 26.8% worked with equids, 36.6% saw farm animals and 9.9% saw exotics (Table 1). Over half (54.9%) worked in small animal‐only practices, and none worked in exotic‐only practices.

**TABLE 1:** Respondents’ practice characteristics (Q.3).

Type of practice	Number of respondents (*n* = 71^a^)	Percentage
Small animal	55	77.5
Farm animal	26	36.6
Equine	19	26.8
Exotics	7	9.9
Small only	39	54.9
Farm only	10	14.1
Equine only	3	4.2
Exotics only	0	0.0

^a^ Respondent 51 of 72 did not complete Q.3.

Chickens were the most commonly seen owned birds (76.4% respondents), followed by psittacines (41.7%) (Table 2). The most commonly seen wild birds were pigeons (22.2%). Practices that only treated small animals saw fewer chickens (odds ratio [OR] = 0.29, 95% confidence interval [CI]: [0.08–0.99], *p* = 0.053), kept ducks (OR = 0.23, 95% CI: [0.08–0.65], *p* = 0.006) and penned/kept game birds (OR = 0.05, 95% CI: [0.01–0.20], *p* < 0.001). Practices that treated farm animals saw more chickens (OR = 13.79, 95% CI: [1.71–111.51], *p* = 0.003), kept ducks (OR = 6.40, 95% CI: [2.18–18.77], *p* < 0.001), turkeys (OR = 6.22, 95% CI: [1.48–26.19], *p* = 0.014) and penned/kept game birds (OR = 10.27, 95% CI: [2.52–41.89], *p *< 0.001).

**TABLE 2:** Species seen by respondents (Q.4).

Species	Number of respondents (*n* = 72)	Percentage
Chickens	55	76.4
Kept Ducks (i.e., not wild)	25	34.7
Turkeys	11	15.3
Kept Geese (i.e., not wild)	11	15.3
Penned/kept game birds (e.g., pheasants, partridges)	16	22.2
Kept psittacines (i.e., not wild)	30	41.7
Kept Columbidae (pigeons/doves, i.e., not wild)	7	9.7
Wild birds—please specify		
Birds of prey	6	8.3
Pigeons	16	22.2
Swans	2	2.8
Wild ducks	2	2.8
Seabirds	7	9.7
Corvidae	4	5.6
Wild geese	2	2.8
Garden birds	4	5.6
Other	3	4.2

The most common range for wild birds seen annually by respondents’ practices was 1–5 birds. The most common range for kept birds was 6–10 birds (Table 3), with some respondents noting that their practices did not accept wild (*n* = 7) or kept (*n* = 5) birds.

**TABLE 3:** Number and percentage of wild and owned birds seen by respondents per year (Q.9–10).

	Number and percentage of respondents seeing wild birds (*n* = 71)	Number and percentage of respondents seeing kept birds (*n* = 72)
None	6 (8.5%)	2 (2.8%)
None—not accepted at the practice	7 (9.9%)	5 (6.9%)
1‒5	21 (29.6%)	12 (16.7%)
6‒10	11 (15.5%)	17 (23.6%)
11‒15	7 (9.9%)	11 (15.3%)
16‒25	8 (11.3%)	13 (18.1%)
26+	11 (15.5%)	12 (16.7%)

### Confidence in seeing birds

The respondents reported varying confidence levels in seeing birds, with 68.0% selecting ‘not at all confident’ or ‘slightly confident’. Only one respondent reported being ‘very confident’ seeing and treating birds (Table 4). High confidence levels in treating avian species were significantly associated with practices that treated farm animals (OR = 3.50, 95% CI: [1.24–9.92], *p* = 0.020) and exotics (OR = 6.39, 95% CI: [1.13–35.97], *p* = 0.031) and practices that saw chickens (OR = 5.33, 95% CI: [1.11–25.53], *p* = 0.041) and kept ducks (OR = 4.84, 95% CI: [1.69–13.86], *p* = 0.004). Low confidence levels were associated with practices that saw fewer than 16 kept birds per year (OR = 0.31, 95% CI: [0.11–0.87], *p* = 0.033).

**TABLE 4:** Respondents’ confidence in seeing birds (Q.5).

Confidence in seeing birds	Number of respondents (*n* = 72)	Percentage
1—not at all confident	25	34.7
2—slightly confident	24	33.3
3—somewhat confident	18	25.0
4—fairly confident	4	5.5
5—very confident	1	1.3

The 49 veterinarians indicating low confidence (scoring 1–2 on Q.5) were invited to state their reasons (Q.6). Among the 48 responses, the most frequent content words (i.e., excluding function words such as ‘the’) indicated that a perceived lack of experience, knowledge, exposure and education affected their confidence in examining avian species. The word ‘lack’ appeared within five words of ‘experience’ 16 times, ‘knowledge’ 12 times and ‘exposure’ 10 times in the responses. The most common phrases in the responses were ‘lack of experience’ (seven instances) and ‘lack of knowledge’ (five instances) (see Table 5 for further context).

**TABLE 5:** Ten most frequent content words in the responses and example context (Q.6).

Content word—example context	Number of instances of content word in responses
Lack—‘lack of resources’	30
Experience—‘Lack of education and experience’	13
Knowledge—‘no knowledge except anecdotal’	9
See—‘Don't see many birds’	8
Teaching—‘Minimal teaching at vet school’	7
Exposure—‘Lack of clinical exposure’	7
Birds—‘Infrequently see birds and find good resources and support hard to find’	5
Training—‘Insufficient training’	5
University—‘Having more handling opportunities at university would've helped’	4
Avian—‘lack of avian experience’	3

**FIGURE 3:**
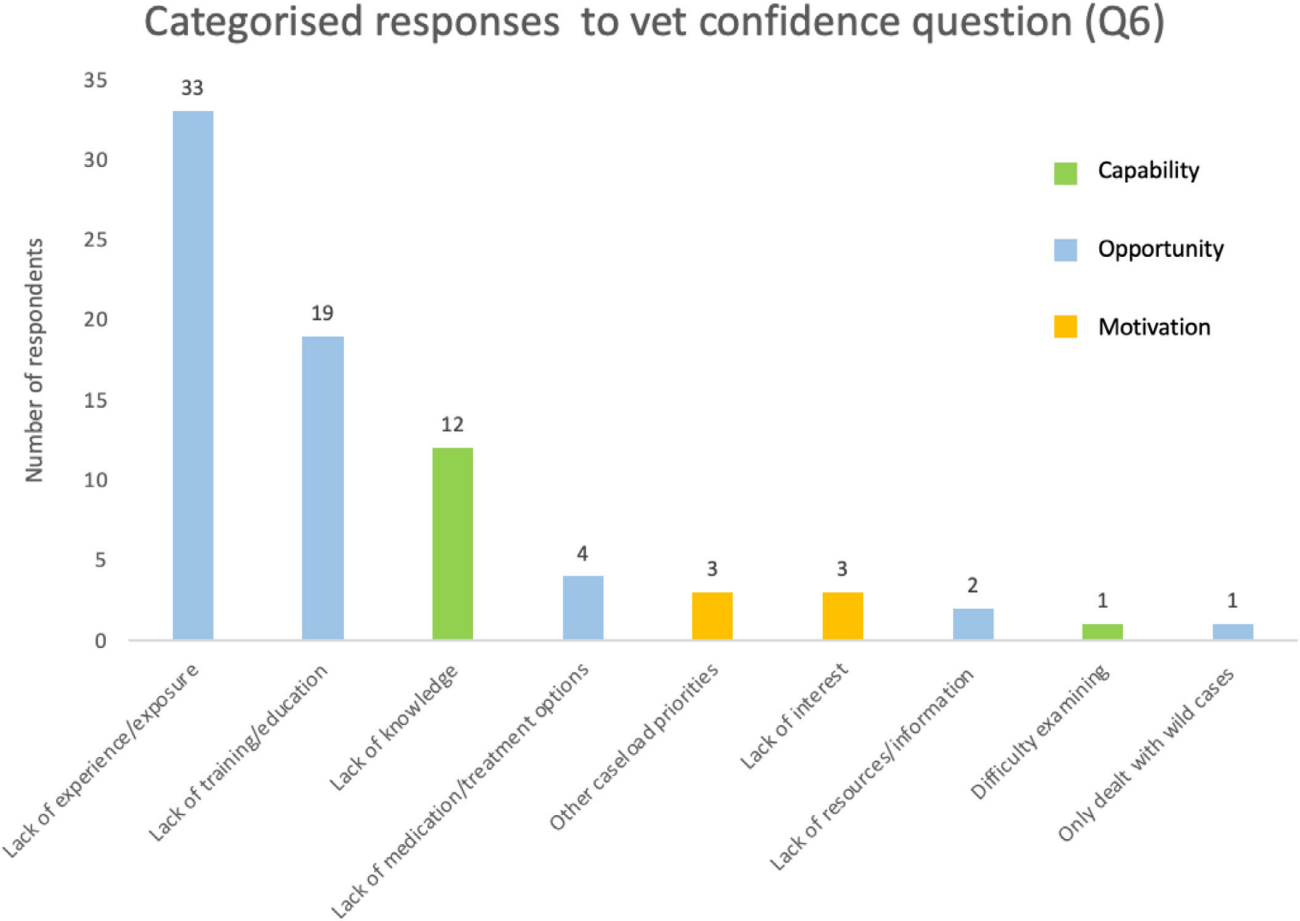
Categorised explanations from respondents who rated their confidence in seeing birds as low (1 or 2) (Q.6).

When we assigned these responses to one or more of nine reasons for low confidence, 24 respondents gave one reason, 19 gave two reasons, four gave three reasons and one gave four reasons. Figure 3 shows the number of responses aligning with each reason, colour coded to reflect COM‐B's capability, opportunity and motivation‐related components.†

Reflecting the large number of responses aligning to COM‐B's opportunity component, under 30% of respondents had undertaken extra bird‐specific training. Of those who specified additional training (18 respondents, detailing 20 training types), 44.4% (*n* = 8) completed a placement; 22.2% (*n* = 4) completed online training; 22.2% (*n* = 4) completed other avian CPD; 5.6% (*n* = 1) carried out own reading/research; 11.1% (*n* = 2) attended lectures; and 5.6% (*n* = 1) completed notifiable disease outbreak training. There was no statistical association between confidence and training (OR = 2.22, 95% CI: [0.76–6.49], *p* = 0.165).

Table 6 is structured according to COM‐B's components and subcomponents and highlights barriers mentioned by respondents in relation to accepting bird cases, particularly those with a possible HPAI infection risk. The final column provides examples from open‐text responses aligning with COM‐B subcomponents.

**TABLE 6:** Barriers to accepting bird cases by COM‐B component (Q.6; Q.20).

Component of COM‐B	Subcomponent of COM‐B	Barrier to a veterinarian accepting a bird case (including if there is a risk of the case being infected with HPAI)	Survey responses
Capability	Psychological	• The clinician does not feel as though they have the knowledge to assess and treat avian species (25%, 12/48 respondents).	• ‘Not enough experience or knowledge’ • ‘Lack of knowledge on conditions and treatment options’ • ‘I don't feel like we see them regularly enough to feel confident with them’. • ‘Wouldn't feel confident with the right steps to take’
	Physical	• The clinician has physical difficulty examining avian species (including concerns regarding safety).	• ‘Difficulty examining […]’ • ‘Uncertainty about how to safely handle and treat the bird’
Opportunity	Social	• The clinician believes support with assessing and treating poultry is hard to find.	• ‘Infrequently see birds and find good resources and support hard to find’.
	Physical	• The clinician feels they have not had appropriate training to deal with an avian species case (40%, 19/48 respondents). • The clinician feels they have little experience with the species, particularly if they are only a small portion of the workload (69%, 33/48 respondents). • They also feel a lack of resources available to refer to for furthering knowledge (4%, 2/48 respondents) and limited drug options (8%, 4/48 respondents) with them being a food producing species.	• ‘Lack of familiarity/caseload/legal medications’ • ‘Lack of avian experience and little focus on avian species in vet school teaching’ • ‘Lack of appropriate medications available’ • ‘Limited exposure and poor university teaching’ • ‘Lack of teaching at uni for backyard poultry—a lot of my notes say postmortem’ • ‘Lack of teaching in university and lack of exposure’ • ‘Very limited training at university and have not done CPD since qualified’
Motivation	Automatic	• The clinician has concerns about a HPAI case and human health.	• ‘Public health risks to myself and the team’ • ‘I would be worried about zoonotic risk and lack of appropriate PPE within the practice’
	Reflective	• The clinician does not perceive avian medicine as a particular area of interest (6%, 3/48 respondents). • The clinician has concerns over loss of use of the practice and the consequences from senior staff members. • The clinician has concerns regarding welfare of other avian species on practice premises.	• ‘Not my main area of interest’ • ‘Also, no interest in avian medicine as not currently useful’. • ‘We simply haven't the facilities to examine a bird outside and if practice was shut down boss would not be impressed’ • ‘Puts the rest of the practice at risk’ • ‘Practice based on farm with laying hens’

Abbreviation: HPAI, highly pathogenic avian influenza.

### Sources of avian influenza information

Of the 72 respondents, 65 (90.3%) were aware of the 2021–2022 and 2022–2023 HPAI outbreaks and associated control measures (Q.11). Of those who provided examples of where they obtained HPAI information (65/72), the most common source (72.3%) was ‘regulatory body communication (e.g., Defra, APHA and Department of Agriculture, Environment and Rural Affairs (DAERA))’, followed by communication within their veterinary practice (49.2%) and ‘British Veterinary Association and their subgroups (BSAVA and British Veterinary Poultry Association (BVPA))’ (32.3%) (Table 7).

**TABLE 7:** Veterinarians’ sources for information regarding the 2021‒2022 and 2022‒2023 highly pathogenic avian influenza outbreaks and the control measures implemented on poultry (Q.12).

Source of information for veterinarians	Number of respondents (*n* = 65)	Percentage
Social media	17	26.2
Poultry register communication	1	1.5
Veterinary Journal	9	13.8
Regulatory body	47	72.3
British Veterinary Association and subgroups (BSAVA and BVPA)	21	32.3
Communications within your practice	32	49.2
Poultry keeper magazine subscription	1	1.5
Farming, game, conservation, animal welfare organisations (e.g., RSPCA, NFU, BASC and GWCT)	4	6.2
Word of mouth	3	4.6
Veterinary school	2	3.1

Abbreviation: BASC, British Association for Shooting and Conservation; BSAVA, British Small Animal Veterinary Association; BVPA, British Veterinary Poultry Association; GWCT, Game and Wildlife Conservation Trust; NFU, National Farmers Union; RSPCA, Royal Society for the Prevention of Cruelty to Animals.

Of 70 responses to Q.23 regarding where veterinarians believed their clients sourced HPAI‐related information (Table 8), social media was the most common (84.3%), followed by news/television and word of mouth (both 44.3%). The respondents could choose multiple options.

**TABLE 8:** Source of information for bird‐keeping clients (Q.23).

Source of information for bird‐keeping clients	Number of respondents (*n* = 70)	Percentage
Social media	59	84.3
Poultry register communication	10	14.3
Your practice/veterinarians	11	15.7
Poultry magazines	16	22.9
News and television	31	44.3
Other internet sources	29	41.4
Word of mouth	31	44.3
Unsure	3	4.3

### Dealing with suspected avian influenza cases

Most respondents who outlined clinical signs of HPAI in Q.15 (68/72 respondents) identified mortality (63.2%) and respiratory signs (54.4%), with fewer identifying cyanosis/discolouration (16.2%), ocular and/or nasal discharge (14.7%), lethargy (16.2%), drop in egg production (13.2%) and swollen head (10.3%). Few mentioned neurological signs (5.9%), diarrhoea (2.9%), reduced feed and/or water intake (4.4%), coughing (2.9%), sneezing/snicking (5.9%) and fever (2.9%). Three respondents were ‘unsure’.

Highlighting capability and opportunity‐related barriers, most respondents (60/72) lacked confidence in ruling out avian influenza as a differential diagnosis. Six had previous direct involvement with HPAI cases, mostly in wild birds, although one respondent reported dealing with a confirmed mammalian case (Table 9).

**TABLE 9:** Respondents’ descriptions of their involvement with highly pathogenic avian influenza cases (Q.17‒18).

Description
‘Sudden death in domestic bird of prey confirmed to be avian influenza’
‘Several herring gulls and a mute swan in previous practice … which were suspected to have AI. All euthanased outside the practice’
‘Wild bird brought in my (sic) member of public tested positive for bird flu on lateral flow penside antigen (SNAP) test. Euthanased’
‘A seal pup that tested positive for avian influenza. I only took the blood sample and nasal swab’

Most (63.9%) respondents would not refuse to see a potentially HPAI‐infected bird (Q.19). For those who would (36%), most elaborated in Q.20 with opportunity‐related barriers, including practice protocol and biosecurity concerns (e.g., lack of appropriate personal protective equipment (PPE)^25^) (both 19.2%), zoonotic risk (11.5%), concerns regarding practices being shut down (11.5%) or apprehension about being stuck on farms (3.8%). Capability‐related reasons included a lack of confidence and/or knowledge (19.2%).

The survey included three true/false questions (Q.21) designed using a BVA guidance document for veterinarians (Table 10).^26^ Most respondents answered the first two questions correctly, but there was discrepancy in the final true/false question to which only 51.4% correctly stated ‘false’ as appropriately housed backyard poultry kept with good biosecurity are lower risk and can be triaged over the phone to rule out respiratory disease before entering practice premises.

**TABLE 10:** Responses to true/false questions in Q.21.

Question	Respondents answering correctly	Respondents answering incorrectly
When the risk of highly pathogenic avian influenza is high, wild birds and un‐housed pet birds should be assessed (including initial examination) outside of the veterinary practice. **True**/F'alse	94.4% (68)	5.6% (4)
For both regulatory and welfare reasons, veterinarians must provide emergency care (first aid including euthanasia) to all sick and injured birds, including wild birds. **True**/False	91.7% (66)	8.3% (6)
When the risk of highly pathogenic avian influenza is high, backyard poultry can never be seen inside the veterinary practice. True/**False**	51.4% (37)	48.6% (35)

*Note*: Correct answers to these questions are highlighted in bold.

When asked about advising clients who suspected HPAI in their flock (Q.22), most respondents mentioned instructing them to contact the APHA (70.0%; *n* = 49), Defra (54.3%; *n* = 38) and/or the regional veterinary inspector (22.9%; *n* = 16). Some would advise clients to bring the bird into practice (7.1%; *n* = 5) or would visit their site (22.9%; *n* = 16). Twelve respondents (17.1%) were unsure how to advise.

### Housing order compliance and vaccination

Overall, respondents believed that clients struggled with housing order measures (Q.24), rating their ability to comply as ‘impossible’ 16.9% (*n* = 12), ‘very difficult’ 42.3% (*n* = 30) and ‘okay’ 39.4% (*n* = 28). Over 60% assumed that there would be no client demand for HPAI vaccination (Q.25), with the most common responses regarding the willingness to pay per vaccine dose (Q.26) being £2.51–5.00 (22.5%; *n* = 16), £5.01–10.00 (19.7%; *n* = 14) and £10.01–15.00 (19.7%; *n* = 14).

### Views on current HPAI control measures

Most respondents were unsure whether existing control measures for HPAI were fit for purpose (69.4%, 50 respondents), with 26.4% (*n* = 19) believing that they were and 4.2% (*n* = 3) saying that they were not (Q.13). In Q.14, some respondents elaborated, citing welfare concerns surrounding HPAI measures:

I'm concerned that the facilities my clients have to house birds for months on end during outbreaks negatively impacts the birds' welfare despite being fit for use when the birds can be free range.

As Table 6 indicates, concerns about practices being closed by the regulatory body if a confirmed HPAI case was seen and a desire for clearer communication from the APHA on reporting suspected cases were expressed by some respondents:

We simply haven't the facilities to examine a bird outside and if practice was shut down boss would not be impressed.

It would be helpful to have better communication from APHA (sic) as to what to do or how to manage a suspected case (I reported a possible case previously and the communication was horrendous …).

Potential enablers for improved HPAI control identified in the open‐text responses included the following:

Realistic standards for backyard poultry keepers with limited funds and/or manpower.

Vaccination of commercial poultry [because] housing measures and culls do not stop wild bird spread and promote poor poultry welfare.

Not have farm vets who maybe see one bird a year going out on surveillance visits!

Backyard poultry keepers struggle to meet the same housing requirements as those placed on commercial keepers. We need to vaccinate commercial birds.

Addressing these barriers or implementing respondents’ suggestions would involve policy and regulatory changes beyond the scope of the individual and practice‐level barriers highlighted in Table 6.

## DISCUSSION

This study investigated GP veterinarians’ confidence in treating avian species and their knowledge and opinions on avian influenza control using COM‐B to identify key barriers to addressing challenges. The use of similar questions to those previously asked allows some comparisons to be drawn with our 2023 study.^15^ With a greater emphasis on GP‐only veterinarians (who do not focus on avian species but will see them if asked), confidence levels were much lower than those reported in the 2023 survey, which included some respondents from poultry practices (57% rated themselves as fairly/very confident in seeing birds compared with 6.8% in this study).

Almost 60% of the GP veterinarians in this study thought compliance with housing order measures was ‘impossible’ or ‘very difficult’ for their clients compared to only 20% of those in the 2023 study, where 10 of 26 respondents had large‐scale commercial clients^15^ (Table 11). As difficulties in complying with housing orders can reduce biosecurity,^11^ GP veterinarians may find themselves increasingly likely to triage avian influenza cases during major HPAI outbreaks.

**TABLE 11:** Comparison of veterinarian opinions on ease of housing order compliance between this survey and the previous 2023 study.^15^

	Impossible (%)	Very difficult (%)	Okay (%)	Easy (%)	Very easy (%)
Veterinary survey 2023, *n* = 26	0 (0)	5 (19.2)	13 (50)	6 (23.1)	2 (7.7)
General practice veterinary survey (this survey Q.24), *n* = 71 (of 72, one respondent did not complete this question)	12 (16.9)	30 (42.3)	28 (39.4)	1 (1.4)	0 (0)

### Application of the COM‐B model

Building on the use of COM‐B to explore how human behaviour affects animal health,^22^, ^23^, ^24^, ^25^ we have identified multiple capability, opportunity and motivation‐related barriers that currently underpin low levels of confidence in (or capacity for) treating avian species and dealing with suspected HPAI cases. Importantly, many of the identified barriers cannot be addressed via individual‐level behaviour change but require national‐level initiatives, including clear government advice, veterinary governing body guidance and engagement with large practice and charity groups. Some of the enablers, meanwhile, reflect misunderstandings about potential efficacy; notably, the assumption that vaccinating commercial poultry would help to prevent HPAI spreading to backyard flocks or reduce the need for housing orders. Drawing on Michie et al.’s behaviour change wheel,^18^ we highlight ‘intervention functions’ (education, training, enablement) and ‘policy categories’ (guidelines, regulation, fiscal measures) with potential to support GP veterinarians to increase capability, opportunities and motivation for treating pet poultry and manage HPAI‐related threats with greater confidence.

#### Capability

Despite backyard poultry keepers often contacting GP veterinarians if they suspect HPAI,^8^, ^9^, ^10^, ^11^, ^13^ many respondents identified significant capability barriers to seeing avian cases and over 80% could not confidently rule out avian influenza as a differential diagnosis. In 2021–2022, 31.9% (39/119) of HPAI outbreaks from 1 October 2021 to 18 July 2022 were reported in ‘backyard’, ‘small’ or ‘smallholder’ flocks.^29^, ^30^ Therefore, it is imperative that GP veterinarians are confident about triaging birds and aware of HPAI reporting procedures.

To tackle capability‐related barriers, it is necessary to address confusion in response to the true/false question (Q.21) ‘When the risk of Highly Pathogenic Avian Influenza is high, backyard poultry can never be seen inside the veterinary practice’. As the BVA Avian Influenza guidance for veterinarians states,^26^ if birds are appropriately housed and owners are practising good biosecurity, extensive measures such as examination outside the practice with appropriate PPE are unlikely to be necessary if there is no prior clinical suspicion of the disease. As many respondents were unaware of this guidance,^26^ there is a need for ‘intervention functions’, including increased education and training opportunities, reinforced with clearer national‐level HPAI‐related regulations and guidelines. These could include ‘enablement’ strategies similar to the risk assessment notes developed by ruminant experts handling the 2024–2025 UK Bluetongue outbreak.^31^ HPAI‐focused risk assessment notes could include seeing suspect cases in situ where possible, creating cost‐effective outdoor isolation facilities (where sick or injured avian species could be appropriately assessed and managed), carrying appropriate PPE, deploying biosecurity measures to manage HPAI‐related risk (including guidelines on storing carcasses while awaiting government intervention) and clear procedures for reporting suspected HPAI cases (including contact details, human health protection teams and fallen stock contractors).

For veterinarians who lack the confidence or facilities to accept avian species, enhanced regulations (or improved implementation of existing guidance) should emphasise requirements for referral arrangements with nearby practices to prevent veterinarians from refusing to see or refer birds on the grounds that they lack confidence or capability.^26^, ^32^ To avoid conflicting advice when veterinarians report suspected HPAI cases,^15^ enhanced national guidelines should clarify these procedures.

#### Opportunity

This study identified a lack of exposure to, or experience with, birds (69% of respondents) as the main cause of low confidence in seeing avian species or identifying suspected HPAI cases. The respondents often linked this to limited opportunities to study avian species, with 25% of those scoring low confidence reporting a general lack of knowledge and 40% reporting a lack of formal training.

While these findings echo previous observations that limited experience with exotic species creates barriers to treating them,^33^ confidence with avian species will only increase with greater exposure. Wild birds present an ideal opportunity to gain experience in handling and examining avian species and are more appropriate than euthanasia for all wild birds brought to practices regardless of condition.^32^

With appropriate fiscal measures, national‐level initiatives supporting further avian‐related training at veterinary school, coupled with CPD in backyard poultry medicine and HPAI‐related public health risks, would support practices in dealing with pet poultry during HPAI outbreaks. CPD opportunities could also cover clinical examination procedures, identifying common presentations and performing first aid treatment, including euthanasia.^8^ Existing online and in‐person courses aimed at veterinarians who lack confidence with poultry could potentially be scaled‐up to provide such training.^12^, ^34^

#### Motivation

Echoing research identifying low interest levels as a barrier to developing confidence with exotic species,^33^ three respondents reported a lack of interest in seeing birds. Motivation‐related barriers also reflected wider biosecurity concerns and apprehension about practices potentially having to close if a suspected HPAI case was seen on premises. When respondents were asked if they would refuse to see a bird if it might be infected with HPAI, five stated that this was their practice's policy, with one writing ‘Because main office of corporate say so’ (respondent 72). There is an urgent need for national‐level guidelines to inform veterinary practices of the consequences to their site in the event of a confirmed HPAI case, along with disinfection protocols for re‐opening practices. Such guidance should also stress the obligation of on‐duty veterinarians to provide emergency first aid to any species until a more appropriate veterinary service accepts responsibility.^35^

On an individual scale, the growing global focus on H5N1 may encourage GP veterinarians to develop greater interest in poultry diseases with the potential for transmission to and between mammals^1^, ^6^, ^7^, ^36^, ^37^, ^38^ that they work more closely with.^39^ With increasing reports of livestock, small carnivores (including dogs, cats and ferrets) and other mammals becoming infected,^1^, ^2^, ^3^, ^4^, ^5^, ^6^, ^7^, ^40^, ^41^, ^42^, ^43^, ^44^ they will need greater familiarity with the clinical signs of H5N1 in these species to rule it out as a differential diagnosis and facilitate prompt reporting.^39^ As cats are more susceptible to HPAI than dogs, particular attention should be paid to those with a history of exposure to wild birds and/or a raw meat or milk diet^45^ or clinical signs, including neurological issues, fever, weight loss, tachycardia, laboured breathing, tremors, hypersensitivity to stimuli, seizures, respiratory and cardiac arrest and ultimately death.^44^ Dogs show milder signs, including fever, anorexia, conjunctivitis, laboured breathing, coughing and diarrhoea.^40^, ^45^

### Study limitations

The survey required a device with an internet connection and a degree of technical literacy to complete and did not capture variations in avian‐related teaching received by respondents trained in Great Britain and abroad. The findings are based on responses from just 72 respondents, and it is possible that more confident veterinarians may be over‐represented. Some respondents’ answers to Q.21 may reflect their practices' policies rather than the BVA guidance.

## CONCLUSION

In this study, 63.2% of GP veterinarians identified mortality as a clinical sign for avian influenza, and 54.4% described respiratory disease. To adequately control HPAI, small animal veterinarians, who are statistically less likely to have confidence seeing avian species than those in other veterinary sectors, must be aware of the clinical signs. If H5N1 cases increase in domestic mammalian species, further research should assess how veterinarians have engaged with the disease and whether this has influenced their confidence in seeing backyard poultry.

Recent infections in barn cats, along with human cases in dairy and poultry farm workers in the United States,^2^ have intensified concern about the potential public health challenges posed by HPAI.^3^, ^5^, ^38^ In Great Britain, the 2024–2025 avian influenza season was associated with 81 H5 cases in poultry/other captive birds and one in sheep.^7^, ^46^ As the first point of contact for many small‐scale poultry keepers, GP veterinarians play a critical role in triaging, reporting and controlling HPAI. Behaviour change frameworks like COM‐B can facilitate the identification of interventions with the potential to address barriers to accurate HPAI diagnosis and reporting in Great Britain and beyond, although these often require national‐level rather than (or in addition to) individual‐level action. Our findings point to a combination of motivation‐related barriers at the individual level and competence/opportunity‐related barriers at the level of the veterinary community and wider national policy and regulatory frameworks.

## AUTHOR CONTRIBUTIONS

Sol Elliott, Sarah Jewitt, Emma McClaughlin, Matthew Smallman‐Raynor, Sioux Fisher and Rachael Tarlinton conceived and designed the project. Sol Elliott acquired the data. Sol Elliott, Sarah Jewitt, Emma McClaughlin, Matthew Smallman‐Raynor and Sioux Fisher analysed and interpreted the data. Sol Elliott, Sarah Jewitt, Emma McClaughlin, Matthew Smallman‐Raynor, Michael Clark and Rachael Tarlinton wrote the paper.

## CONFLICT OF INTEREST STATEMENT

The authors declare no conflicts of interest.

## ETHICS STATEMENT

Ethics permission was granted by the School of Geography, University of Nottingham (Ref. 010).

## Supporting information



Supporting Information

## Data Availability

The data that support the findings of this study are available from the corresponding author upon reasonable request.

## Footnotes

* Unfortunately, we had no respondents from Northern Ireland.

†
*Note*: The totals add up to more than 48 because several respondents gave more than one reason for low confidence.
