# Supplementary material for: General practice veterinarians’ attitudes towards avian influenza: A COM‐B analysis of barriers to backyard poultry treatment
Source: Vet Rec. 2025 Dec 22;198(6):e237–47. doi: 10.1002/vetr.70173 (PMC12983996; doi:10.1002/vetr.70173)
Supplement: Supplementary file 1 — Supporting Information [file VETR-198--s001.docx]

**Appendix 1: Survey information and flow diagram**

**Understanding GP Vets' Knowledge of Highly Pathogenic Avian Influenza**

This survey is being conducted by the School of Veterinary Medicine and Science at the University of Nottingham. We are seeking to understand general practice vets’ understanding of Highly Pathogenic Avian Influenza control measures. With 2021-2022 seeing the largest Highly Pathogenic Avian Influenza outbreak in the UK, we are keen to explore veterinarians’ knowledge of the disease, how they interpret the guidance and regulations put in place during an outbreak as well as their overall confidence levels when presented with avian species. The study has ethical approval from the School's clinical ethics panel. All responses to the survey will be held on a secure database at the University of Nottingham and will be anonymised. The collated results of the survey may be published in academic journals or shared with government, veterinary or poultry industry bodies but will not be used for commercial purposes. Individual responses will not be identifiable in these reports. If you would like to receive a copy of the results of the survey you can provide an email address for this to be sent to. If you wish for your responses to be removed or to view the data held from your responses at any stage please contact the researchers involved in this survey.

Note this survey is **NOT** for commercial poultry vets whose caseload is predominantly poultry focused.

1. **Are you predominantly a general practice vet, working in private practice, who would describe their case load as “not predominantly avian focused” but may see pet or backyard poultry if requested**. Yes; No.
2. **First section of your practice postcode** (e.g. if your postcode is LE12 5RD, please type LE12)
3. **What type of practice do you work in? Select all that apply**. Small animal; Farm animal; Equine; Exotic.
4. **In the past 12 months, which of the following avian species have been treated by your practice? Select all that apply**. Chickens; Kept Ducks (i.e. not wild); Turkeys; Kept Geese (i.e. not wild); Penned/kept game birds (e.g. pheasants, partridges); Kept Psittacines (i.e. not wild); Kept Columbidae (pigeons/doves i.e. not wild); Wild Birds – please specify below; Other.
5. **How would you rate your confidence seeing and treating birds?** 1 - not at all confident; 2 - slightly confident; 3 - somewhat confident; 4 - fairly confident; 5 - completely confident
6. **If you scored 1 or 2, what do you think the possible reasons are for this?**
7. **Have you taken any bird-specific training (excluding vet school unless you undertook electives/EMS placements with an avian focus)?** Yes; No.
8. **If you answered yes, please specify the nature of training and time spent doing this**
9. **How many *owned/kept birds* does your practice see in a typical year?** None - not accepted at the practice; None, 1-5; 6-10; 11-15; 16-25; 26+
10. **How many *wild birds* does your practice see in a typical year**? None - not accepted at the practice; None, 1-5; 6-10; 11-15; 16-25; 26+
11. **Are you aware of the control measures for poultry implemented during the 2021-2 and 2022-3 outbreaks of Highly Pathogenic Avian Influenza (e.g. housing measures for poultry and other captive birds, 3Km protection zones and 10Km surveillance zones around infected premises.)?** Yes; No
12. I**f yes where did you get this information? Select all that apply**. Social media; Poultry register communication; Veterinary Journal; Regulatory body communication (e.g. Defra, the APHA, DAERA); British Veterinary Association and their sub-groups (BSAVA, BVPA); Communications within your practice; Poultry keeper magazine subscription; Farming, game, conservation, animal welfare organisations (e.g. RSPCA, NFU, BASC, GWCT); Other - please specify below.
13. **Do you consider the control measures implemented during the 2021-2 and 2022-3 outbreaks of Highly Pathogenic Avian Influenza to be fit for purpose?** Yes; No; Unsure.
14. **If** **you answered no, what other options do you consider to be of value in aiding Highly Pathogenic Avian Influenza control?**
15. **What** **clinical signs would make you put Highly Pathogenic Avian Influenza on your differential diagnosis list?**
16. **Are you confident that you could rule out Highly Pathogenic Avian Influenza as a differential?** Yes; No
17. **Have you ever had direct involvement in a case of Highly Pathogenic Avian Influenza**? Yes; No
18. **If you answered yes please give details of the nature of the case (or cases). Please do not include personal client information in your response**
19. **Would the possibility of a case being infected with Highly Pathogenic Avian Influenza result in you refusing to see the bird(s)?** Yes; No
20. **If you answered yes please explain why**.
21. **Please answer True or False to the following questions**:-

- **When the risk of Highly Pathogenic Avian Influenza is high, wild birds and un-housed pet birds should be assessed (including initial examination) outside of the veterinary practice**. True; False.
- **For both regulatory and welfare reasons, vets must provide emergency care (first aid including euthanasia) to all sick and injured birds, including wild birds**. True; False.
- **When the risk of Highly Pathogenic Avian Influenza is high, backyard poultry can never be seen inside the veterinary practice**. True; False

1. **How would you advise a client who contacted you about a suspected case of Highly Pathogenic Avian Influenza? Select all that apply**. Ask client to bring the bird to the practice premises; Inform client that you will arrange a site visit; Instruct client to phone regional veterinary inspector; Instruct client to contact the APHA; Instruct client to contact the DEFRA helpline; Instruct client to contact a poultry veterinary practice; Unsure
2. **Where do you believe your bird-keeping clients are getting most of their Highly Pathogenic Avian Influenza information from? Select all that apply**. Social Media; Poultry register communication; Your practice/vets; Poultry magazines; News and television; Other internet sources; Word of mouth; Other - please specify below; Other
3. **How easy do you think it is for the majority of your bird-keeping clients to implement the mandatory housing order when required to do so?** Impossible (1); Very difficult (2); Okay (3); Easy (4); Very easy (5)
4. **Do you think there would be a demand for vaccinating against Highly Pathogenic Avian Influenza within your client base?** Yes; No.
5. **If an individual vaccine were to become available for Highly Pathogenic Avian Influenza which may mean that some of the control measures could differ from the current methods used (e.g. mandatory housing), how much would you think clients would be willing to pay per dose, not including other veterinary fees?** Nothing; £0.01 - £2.50; £2.51 - £5.00; £5.01 - £10.00; £10.01 - £15.00; £15.01 - £20.00; Over £20
6. **If you have any other comments about Highly Pathogenic Avian Influenza, please use the space below**.
